# Azithromycin in crisis: antimicrobial resistance, diagnostics gaps, and vaccine-linked stewardship for enteric infections in low- and middle-income countries

**DOI:** 10.1016/j.ijregi.2026.101008

**Published:** 2026-09-11

**Authors:** Jobin John Jacob, Madhumathi Irulappan, Kamini Walia, Yamuna Devi Bakthavatchalam, Balaji Veeraraghavan

**Affiliations:** 1 Department of Clinical Microbiology, Christian Medical College, Vellore, India; 2 Division of Descriptive Research, Indian Council of Medical Research, New Delhi, India

**Keywords:** Azithromycin, *S. Typhi*, *S. Paratyphi*, *Shigella* sp, Non-typhoidal *Salmonella*, *Campylobacter*

## Abstract

**Background:**

Azithromycin remains an important oral treatment option for enteric infections in low- and middle-income countries (LMICs), where rising fluoroquinolone resistance, limited diagnostic capacity, and restricted access to parenteral therapy have encouraged widespread empirical use. Increasing azithromycin resistance among *Shigella* sp., typhoidal and non-typhoidal *Salmonella*, and *Campylobacter sp.*, however, threatens its clinical utility.

**Objectives:**

This Perspective examines azithromycin resistance as a health-system challenge rather than solely a microbiological phenomenon. Diagnostic gaps, prolonged empirical prescribing, over-the-counter availability, and inadequate antimicrobial stewardship contribute to sustained selective pressure. The drug’s prolonged half-life, extensive tissue and intracellular penetration, and sustained intestinal exposure may further facilitate resistance selection. Importantly, resistance mechanisms, susceptibility patterns, and clinical-microbiological relationships differ substantially among enteric pathogens, limiting the value of uniform treatment strategies. The Perspective highlights diagnostic stewardship, pathogen-specific surveillance, locally relevant susceptibility interpretation, and vaccination as complementary approaches to reducing unnecessary azithromycin exposure.

**Conclusion:**

Preserving azithromycin requires coordinated health-system interventions that integrate accessible diagnostics, pathogen-directed antimicrobial stewardship, resistance surveillance, regulation of antibiotic access, and vaccination. Such an approach is particularly important in LMICs, where azithromycin remains one of the few accessible oral options for severe enteric infections.

## Highlights

• Azithromycin resistance is rising among major enteric pathogens in low- and middle-income countries.
• Mechanisms and clinical impact vary across *Shigella, Salmonella*, and *Campylobacter*.
• Diagnostics and pathogen-directed stewardship are critical interventions.
• Surveillance is key to preserving azithromycin efficacy.

## Introduction

Azithromycin has become one of the most commonly prescribed oral agents for bacterial enteric infections, largely driven by increasing fluoroquinolone resistance and the need for effective outpatient antimicrobial therapy. Its favorable pharmacokinetic profile, including extensive tissue and intracellular penetration and convenient dosing, has encouraged widespread empirical use. This has been particularly pronounced in pediatric populations and resource-limited settings, where diagnostic capacity and access to parenteral therapy are limited. However, resistance is emerging among key enteric pathogens, notably *Shigella*, typhoidal *Salmonella*, non-typhoidal *Salmonella* (NTS), and *Campylobacter*, with reports of elevated minimum inhibitory concentrations (MICs) and clinical failure threatening its clinical effectiveness [[1], [2], [3], [4]].

Azithromycin is classified by the World Health Organization as an antibiotic with a high potential to promote antimicrobial resistance (AMR). It is included in the Watch category of the World Health Organization Access–Watch–Reserve classification, for which restricted use and close monitoring are recommended due to an elevated risk of resistance selection at the individual and population levels [5]. Consistent with this designation, increasing and often indiscriminate azithromycin use has been accompanied by rising resistance among key enteric pathogens in recent years, particularly in South and Southeast Asia [6].

Beyond pathogen-specific resistance patterns, azithromycin exhibits important pharmacokinetic–pharmacodynamic liabilities. Its prolonged terminal half-life of more than 50 hours, intracellular and prolonged tissue concentration, and sustained high intestinal exposure generate persistent selective pressure within the gut microbiota, facilitating the emergence and maintenance of macrolide resistance [7]. Furthermore, despite high intracellular accumulation, azithromycin predominantly provides bacteriostatic or suppressive activity, which may be suboptimal in infections characterized by a high bacterial burden or rapid epithelial cell-to-cell spread [8]. This pharmacokinetics and pharmacodynamics (PK/PD)–pathogenesis discordance may contribute to clinical failure despite *in vitro* susceptibility and underscores the limitations of MIC-based interpretations for guiding therapy.

Importantly, azithromycin use for enteric infections remains off label and is not included in Food and Drug Administration (FDA)-approved labeling [9]. Although it addresses an important therapeutic gap, its expanding use risks promoting resistance and compromising long-term effectiveness. These observations underscore the need for stewardship-focused strategies that emphasize pathogen-directed therapy over routine empiric use. This commentary argues that azithromycin resistance is more than a microbiological trend. It reflects broader health system weaknesses, including gaps in diagnostics, suboptimal stewardship, and underinvestment in preventive measures such as vaccination.

## Azithromycin resistance as a health systems problem

In many low- and middle-income countries (LMICs), antibiotic prescribing is driven more by structural constraints than by pathogen-specific diagnoses. High patient volumes, limited laboratory capacity, fragmented health care delivery, and over-the-counter availability converge to make empirical azithromycin use routine. As a result, azithromycin has effectively become a universal oral fallback for febrile and diarrheal illnesses, irrespective of etiology or resistance profile.

In India, azithromycin is the most heavily consumed single-molecule antibiotic, with an estimated 640 million defined daily doses, representing 12.6% of total antibiotic use [10]. Worldwide, widespread and poorly regulated azithromycin use risks undermining gains in infectious disease control. The impact is greatest in LMICs, where limited therapeutic alternatives and rising resistance among enteric pathogens threaten to reverse hard-won public health gains. These trends underscore the urgency of integrating antimicrobial stewardship within broader health system strengthening.

## Azithromycin resistance mechanism in enteric pathogens

Resistance dynamics vary substantially across enteric pathogens (Table 1). In typhoidal *Salmonella*, azithromycin resistance is gradually increasing in South Asia, including India, Pakistan, and Bangladesh, driven by acrB efflux pump mutations and plasmid-mediated *mph*A [1,11,12]. *Shigella* increasingly exhibits high-level, plasmid-mediated macrolide resistance, primarily through *mph*A-mediated macrolide inactivation and *erm*B mediated ribosomal methylation, which strongly correlate with clinical treatment failure [13,14]. Outbreaks of azithromycin-resistant *Shigella* have been reported across South Asia and Africa, often involving strains that are already resistant to fluoroquinolones and third-generation cephalosporins [[14], [15], [16]].

**Table 1:** Azithromycin resistance in major enteric pathogens: implications for clinical care, stewardship, and health systems in LMICs.

Pathogen	Resistance mechanism	Pathogenesis	MIC range (μg/mL)	MIC break point interpretation	Clinical outcome	Stewardship implication	Health systems/policy relevance
Shigella spp.	Plasmid-mediated; (*mphA, ermB, msrE*)	Intracellular colonic epithelium	16-256 (*S. flexneri*)	≥16 resistant; failure correlation	Prolonged dysentery, treatment failure	High-resistance settings: avoid empirical use; pathogen-specific	Routine surveillance; investment for rapid diagnostics
Non-typhoidal Salmonella	Chromosomal: efflux pump Plasmid: *mphA, erm(B)*	Systemic, macrophages	4-32 (up to 64)	Moderate MICs may retain efficacy	Delayed response, relapse	Optimize dosing; reserve for confirmed indications.	MIC-based guidance and differentiated treatment algorithms
Typhoidal Salmonella	AcrB (R717Q/L)	Intestinal invasion/bacteremia	8-64	Elevated; breakpoints revising	Persistent illness, sepsis risk	Escalate only with susceptibility confirmation.	Supports vaccination-led reduction in azithromycin pressure
Campylobacter spp.	23S rRNA (A2075G)	Mucosal jejunum/colon	8-64	≥8 failure; species variation	Prolonged diarrhea, transmission	Discourage usage for uncomplicated diarrhea.	Supports One Health surveillance and non-antibiotic controls
All enteric pathogens (aggregate)	Co-circulation of MDR/XDR strains	Variable	Variable (4-256)	Variable	Poor treatment outcome	Shift from drug-centric to syndrome–pathogen–based stewardship.	Strengthens investment in diagnostics, vaccines, and regulation

LMIC, low- to middle-income country; MIC, minimum inhibitory concentration; rRNA, ribosomal ribonucleic acid.

In contrast, NTS shows more heterogeneous susceptibility; modest MIC elevations, often driven by *mph*A and *erm*B, may still permit clinical efficacy in selected cases [17]. *Campylobacter sp.,* exhibits widespread, largely community-driven macrolide resistance, associated with ribosomal target modifications and efflux mechanisms, frequently limiting the clinical benefit of therapy [18]. Treating these infections as a single entity masks important pathogen-specific differences, promotes inappropriate antibiotic use, and accelerates the emergence and spread of resistance, underscoring the urgent need for pathogen-directed therapy and targeted stewardship.

## Rethinking antimicrobial stewardship for azithromycin

Azithromycin’s widespread use for enteric and respiratory infections, particularly in LMICs, has outpaced diagnostic capacity and surveillance, driving resistance. Conventional stewardship frameworks that target narrow-spectrum agents or hospital-based interventions are insufficient. For azithromycin, such approaches may be ineffective or even harmful, given its role as one of the few orally available options for severe enteric infections.

A more effective approach is pathogen- and syndrome-specific stewardship that considers resistance mechanisms, disease severity, and health care access. This requires moving beyond blanket restrictions toward precision-guided clinical algorithms informed by local resistance data. Lacking such precision, stewardship risks fostering resistance and denying effective treatment. Strengthening regulations, surveillance, and preventive interventions, including vaccination and improved sanitation, is critical to preserving azithromycin’s clinical utility and curbing its contribution to the global AMR crisis.

## Diagnostics gaps as the central driver of azithromycin misuse

The absence of accessible diagnostics is arguably the single most important driver of inappropriate azithromycin use in LMICs. Clinicians in most settings lack reliable tools to distinguish inflammatory bacterial diarrhea from viral or non-invasive diseases. Culture-based susceptibility testing is slow and often unavailable, whereas molecular detection of resistance mechanisms remains largely confined to research settings. This diagnostic gap drives widespread empirical treatment, prolonged ineffective therapy, and sustained transmission of resistant strains [19]. It also undermines stewardship because clinicians cannot safely withhold antibiotics without confidence in an alternative diagnosis. Investments in diagnostics should, therefore, be viewed as antibiotic-preserving interventions rather than optional laboratory enhancements [20]. Integrating enteric pathogen testing into existing febrile illness and sepsis platforms could substantially reduce inappropriate macrolide exposure.

Recent advances in molecular diagnostics have significantly improved the detection of gastrointestinal pathogens. Commercial multiplex panels, including the FilmArray gastrointestinal panel (BioFire Diagnostics, Salt Lake City, UT, USA), the Luminex xTAG gastrointestinal pathogen panel (Luminex Corporation, Toronto, Canada), and the Nanosphere Verigene enteric pathogen test (Nanosphere, Inc., Northbrook, IL, USA), enable the rapid and sensitive detection of bacteria, viruses, and parasites directly from stool. These FDA-approved platforms facilitate timely, pathogen-directed therapy; strengthen antimicrobial stewardship; and can help curb unnecessary azithromycin use in hospital and community settings.

## Beyond minimum inhibitory concentration: clinical–microbiological discordance

Importantly, clinical–microbiological discordance has been documented for azithromycin in typhoidal *Salmonella* [[21], [22], [23]]. In a controlled human infection model of *S. typhi*, azithromycin treatment was associated with prolonged bacteremia and slower fever clearance, despite predicted intracellular drug concentrations exceeding the MIC; however, systemic plasma concentrations remained below the MIC [24]. These findings highlight the limitations of interpreting azithromycin MICs solely in relation to plasma exposure for an intracellular pathogen.

Evidence from shigellosis further demonstrates that *in vitro* susceptibility does not always translate directly into a clinical response [25,26]. In a prospective observational cohort from Bangladesh, decreased azithromycin susceptibility in *S. flexneri* was associated with persistent diarrhea, continued stool culture positivity, and hospitalization [25]. Notably, patients infected with *S. sonnei* experienced poor outcomes even when isolates had a low azithromycin MIC of 4 µg/mL, illustrating that clinical outcomes may not be fully predicted by MIC alone [26]. Together, these observations support the need to interpret azithromycin MICs in conjunction with the pathogen, resistance mechanism, pharmacokinetic–pharmacodynamic characteristics, and clinical outcome rather than relying exclusively on categorical susceptibility.

Interpretation of azithromycin susceptibility is further complicated by differences in the approaches adopted by Clinical and Laboratory Standards Institute (CLSI) and The European Committee on Antimicrobial Susceptibility Testing (EUCAST) [27,28]. CLSI considers azithromycin MICs of ≤16 mg/L as susceptible for the relevant enteric pathogens, whereas EUCAST uses a more cautious approach, indicating that isolates with MICs >16 mg/L or 15-µg disk diffusion zone diameters <12 mm, are likely to harbor azithromycin resistance mechanisms. Although wild-type MIC distributions may vary somewhat between populations, the EUCAST criterion is primarily intended to identify isolates likely to harbor resistance mechanisms rather than to define an equivalent categorical susceptible breakpoint. Consequently, isolates with MICs around the 16 mg/L threshold warrant cautious interpretation because differences in break point criteria may influence reported resistance rates and potentially affect clinical decision-making. These differences highlight the importance of specifying the interpretive standard and break point version when reporting azithromycin susceptibility data and comparing results across studies or surveillance settings.

## Vaccines as AMR interventions

Vaccination represents one of the most effective yet underutilized strategies for reducing antibiotic pressure in enteric infections. By preventing disease, vaccines directly reduce antibiotic consumption and limit the selection of resistant strains in target pathogens and the broader gut microbiome.

Typhoid conjugate vaccines have already demonstrated reductions in febrile illness and antibiotic use in endemic settings [29]. Future *Shigella* and NTS vaccines could have an even greater impact, given the high burden of antibiotic-treated diarrheal disease caused by these pathogens [30]. Despite this potential, vaccine policy discussions rarely incorporate AMR benefits into cost-effectiveness analyses or prioritization frameworks. Explicitly framing vaccines as AMR mitigation tools could accelerate their development, introduction, and sustained financing.

## Surveillance, break points, and policy alignment

Current azithromycin breakpoints and treatment guidelines are largely derived from high-income settings and may not adequately reflect the realities in LMICs. Factors such as malnutrition, delayed health care access, and co-infections amplify the impact of elevated MICs, increasing the risk of treatment failure, even when isolates are categorized as susceptible or intermediate. National AMR action plans should prioritize enteric pathogen-specific surveillance, including routine reporting of azithromycin MIC distributions and resistance trends. Surveillance should extend beyond categorical susceptibility reporting to include longitudinal monitoring of MIC distributions, shifts in MICs over time, and the emergence of high-level resistance among major enteric pathogens. Such data can provide early warning of declining antimicrobial activity before resistance becomes widespread and support the development of locally relevant treatment recommendations.

A One Health approach is equally important because resistant enteric organisms and resistance determinants can circulate across interconnected human, animal, food, and environmental reservoirs [31,32]. Linking AMR surveillance across these sectors can help identify transmission pathways and shared selective pressures that may not be apparent from human clinical surveillance alone. Data-sharing platforms that integrate human, animal, food, and environmental surveillance provide a more comprehensive assessment of the emergence and dissemination of azithromycin resistance. Importantly, locally generated data are essential for guiding empirical therapy and informing context-specific stewardship policies.

## Policy recommendations

Integrated strategies are needed to preserve azithromycin efficacy and curb resistance in enteric infections, particularly in LMICs. We propose five priority actions: (i) recognize azithromycin as a protected, indication-specific antibiotic, (ii) embed enteric pathogen surveillance within national AMR strategies, (iii) scale affordable diagnostics as core stewardship tools, (iv) accelerate vaccine deployment with explicit AMR objectives, and (v) strengthen regulation of over-the-counter macrolide access.

## Conclusion

The convergence of azithromycin resistance in *Shigella*, NTS, typhoidal *Salmonella*, and *Campylobacter* reflects systemic weaknesses rather than inevitable microbial evolution. Without coordinated action across diagnostics, stewardship, surveillance, and vaccination, LMICs face an imminent risk of losing one of the last effective oral agents for severe pediatric enteric infections. Integrating these domains offers a realistic and urgently needed pathway to preserve azithromycin while strengthening health system resilience against AMR.

## Funding

This research did not receive any specific grant from funding agencies in the public, commercial, or not-for-profit sectors.

## Ethical approval

This article is a perspective and does not involve any human participants, clinical data, or animal experiments. Therefore, ethical approval was not required.

## CRediT authorship contribution statement

**Jobin John Jacob:** Conceptualization, Data curation, Writing – original draft, Writing – review & editing. **Madhumathi Irulappan:** Conceptualization, Data curation, Writing – original draft, Writing – review & editing. **Kamini Walia:** Writing – review & editing. **Yamuna Devi Bakthavatchalam:** Writing – original draft, Writing – review & editing. **Balaji Veeraraghavan:** Conceptualization, Writing – original draft, Writing – review & editing.

## Declaration of competing interest

The authors have no competing interests to declare.
